# Supplementary material for: Three-Dimensional Automated, Machine-Learning-Based Left Heart Chamber Metrics: Associations with Prevalent Vascular Risk Factors and Cardiovascular Diseases
Source: J Clin Med. 2022 Dec 12;11(24):7363. doi: 10.3390/jcm11247363 (PMC9782505; doi:10.3390/jcm11247363)
Supplement: Supplementary file 1 [file jcm-11-07363-s001.zip › jcm-2003328-supplementary.pdf]

Table S1. Characteristics of 22 patients selected for intra- and inter-operator variability assessment.

Patient ID	Image quality	FR op A (Hz)	FR op B (Hz)	Category
**1**	Suboptimal	19	17	CVDs
**2**	Suboptimal	20	17	CVDs
**3**	Optimal	21	18	CVDs
**4**	Suboptimal	22	22	Healthy
**5**	Optimal	20	19	CVDs
**6**	Optimal	20	20	VRFs
**7**	Optimal	21	21	CVDs
**8**	Suboptimal	19	19	CVDs
**9**	Optimal	20	20	VRFs
**10**	Suboptimal	18	15	CVDs
**11**	Suboptimal	17	17	Healthy
**12**	Optimal	17	20	VRFs
**13**	Optimal	24	25	CVDs
**14**	Optimal	20	20	Healthy
**15**	Optimal	22	21	CVDs
**16**	Optimal	20	20	CVDs
**17**	Optimal	21	21	VRFs
**18**	Suboptimal	19	19	CVDs
**19**	Optimal	19	19	Healthy
**20**	Optimal	19	19	Healthy
**21**	Optimal	19	19	Healthy
**22**	Optimal	21	21	Healthy

Legend: FR= frame rate**;** Op A= senior operator; Op B= junior operator.

Table S2. Associations between 3D-DHM echocardiographic metrics and vascular risk factors or prevalent cardiovascular disease.

	**Prevalent VRFs**		**Prevalent CVDs**	
**Echocardiographic metrics**	aOR* [95 % CI]	p value	aOR* [95% CI]	p value
**EDVi 3D (per 10 ml/m^2)**	0.98 [0.85-1.13]	0.79	1.44 [1.33-1.57]	<0.001
**ESVi 3D, (per 10 ml/m^2)**	1.02 [0.76-1.35]	0.90	2.31 [1.97-2.71]	<0.001
**LVEF 3D (per 10%)**	0.92 [0.63-1.34]	0.65	0.28 [0.22-0.35]	<0.001
**LAEF 3D (per 10%)**	0.94 [0.77-1.15]	0.58	0.45 [0.39-0.51]	<0.001
**LV mass/m2 (per 10 g/m^2)**	1.03 [0.90-1.18]	0.58	1.50 [1.37-1.64]	<0.001
**LAVi, max (per 10 ml/m^2)**	1.15 [0.96-1.38]	0.11	1.73 [1.55-1.94]	<0.001
**LAVi min (per 10 ml/m^2)**	1.19 [0.87-1.63]	0.26	3.03 [2.48-3.70]	<0.001
**SVi (per 10 ml/m^2)**	0.94 [0.75-1.17]	0.61	0.95 [0.82-1.09]	0.49
**LVGF index (per 10%)**	0.77 [0.51-1.19]	0.25	0.22 [0.16-0.28]	<0.001
**LVM/LVEDV ratio**	1.91 [0.59-6.16]	0.27	0.83 [0.39-1.73]	0.61

Legend: *Adjusted analysis for sex and age; EDVi= end-diastolic volume indexed; ESVi= end-systolic volume indexed; LAEF= left atrial ejection fraction; LAVi= left atrial volume indexed; LVEF= left ventricular ejection fraction, LVGFI= left ventricle global function index, LVM= left ventricular mass, Svi= stroke volume indexed.

Table S3. Intra- (A) and inter-operator (B) reliability assessed by Bland-Altman analysis, Interclass Correlation Coefficients, and Coefficients of Variance.

A

	**Bias**	**LOA**	**ICC**	**CoV**
**EDV**	1.5	14.9	99.5%	2.5 ± 2.1%
**ESV**	-0.5	5.6	99.8%	2.4± 1.8%
**LVEF-3D**	0.5	4.0	97.8%	2.1±1.5%
**SV**	1.9	13.4	98.4%	4.1±3%
**LAVmax**	2.1	21.1	93.6%	5.9±6.5%
**LAVmin**	0.4	11.8	95.7%	8.1±6.2%
**LAEF**	-0.4	13.7	86.2%	7.1±6.3%

B

	**Bias**	**LOA**	**ICC**	**CoV**
**EDV**	5.0	25.6	98.8%	4.3%±3.7%
**ESV**	1.0	15.4	99.2%	5.2%±3.8%
**LVEF-3D**	0.9	8.1	95.4%	4.6±3.6%
**SV**	5.0	26.2	92.2%	8.2±4.5%
**LAVmax**	3.7	18.0	94.9%	7.2±6.1%
**LAVmin**	3.4	10.9	92.9%	11.3±6.3%
**LAEF**	-1.7	14.1	82.5%	6.5±7.1%

Legend: CoV= Coefficient of variance; EDV= end-diastolic volume; ESV= end-systolic volume; ICC= Interclass correlation coefficients; LAEF= left atrial ejection fraction; LAV= left atrial volume; LOA= limits of agreement; LVEF= left ventricular ejection fraction, LVGFI= left ventricle global function index, LVM= left ventricular mass, SV= stroke volume.

Figure S1. 3D-DHM Echocardiographic metrics stratified according to the presence of VRF and CVDs.

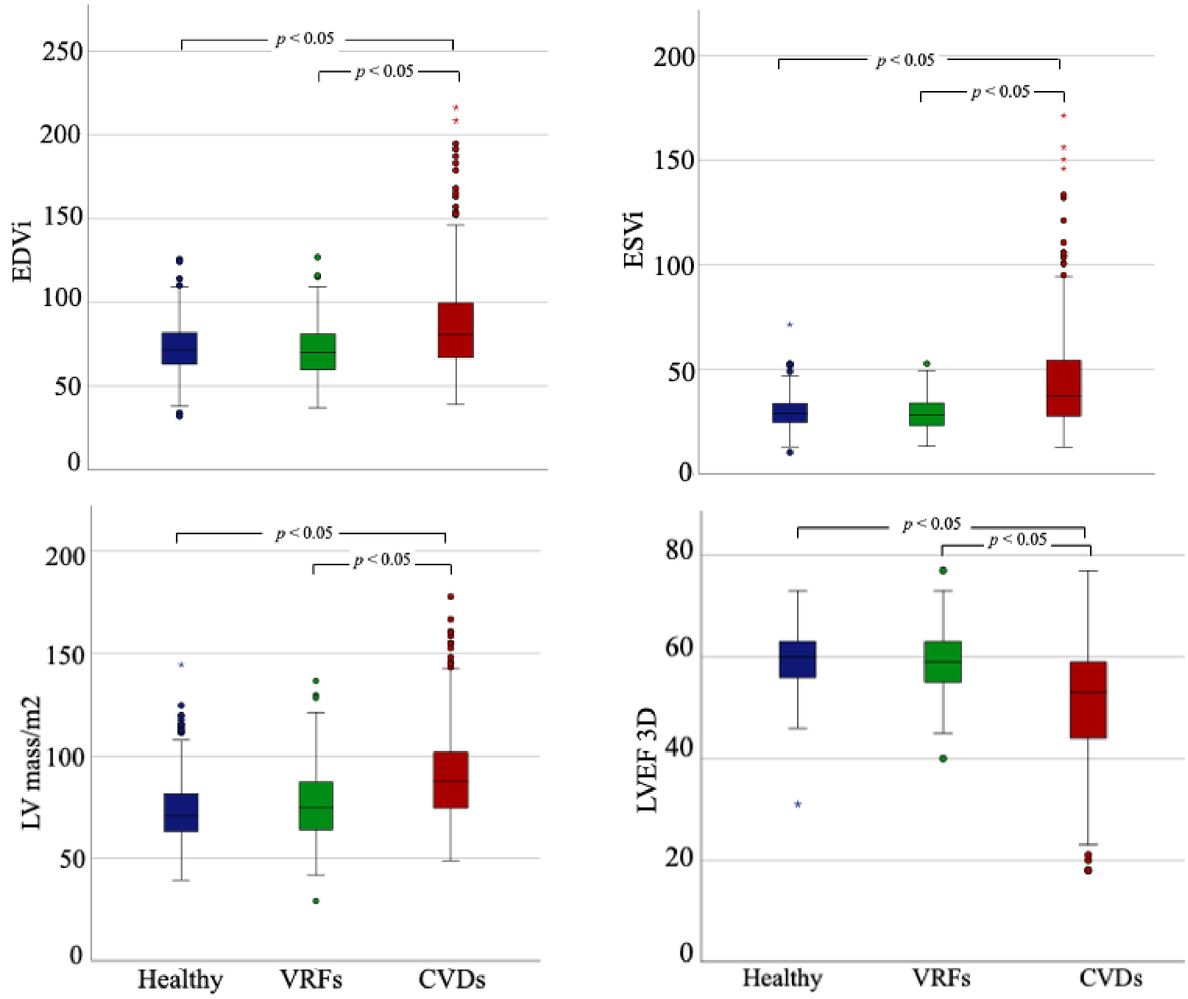

Legend: EDVi= end-diastolic volume indexed; ESVi= end-systolic volume indexed; LVEF= left ventricular ejection fraction.

Figure S2. Intra-operator variability assessed by Pearson's correlation and Bland-Altman Plot.

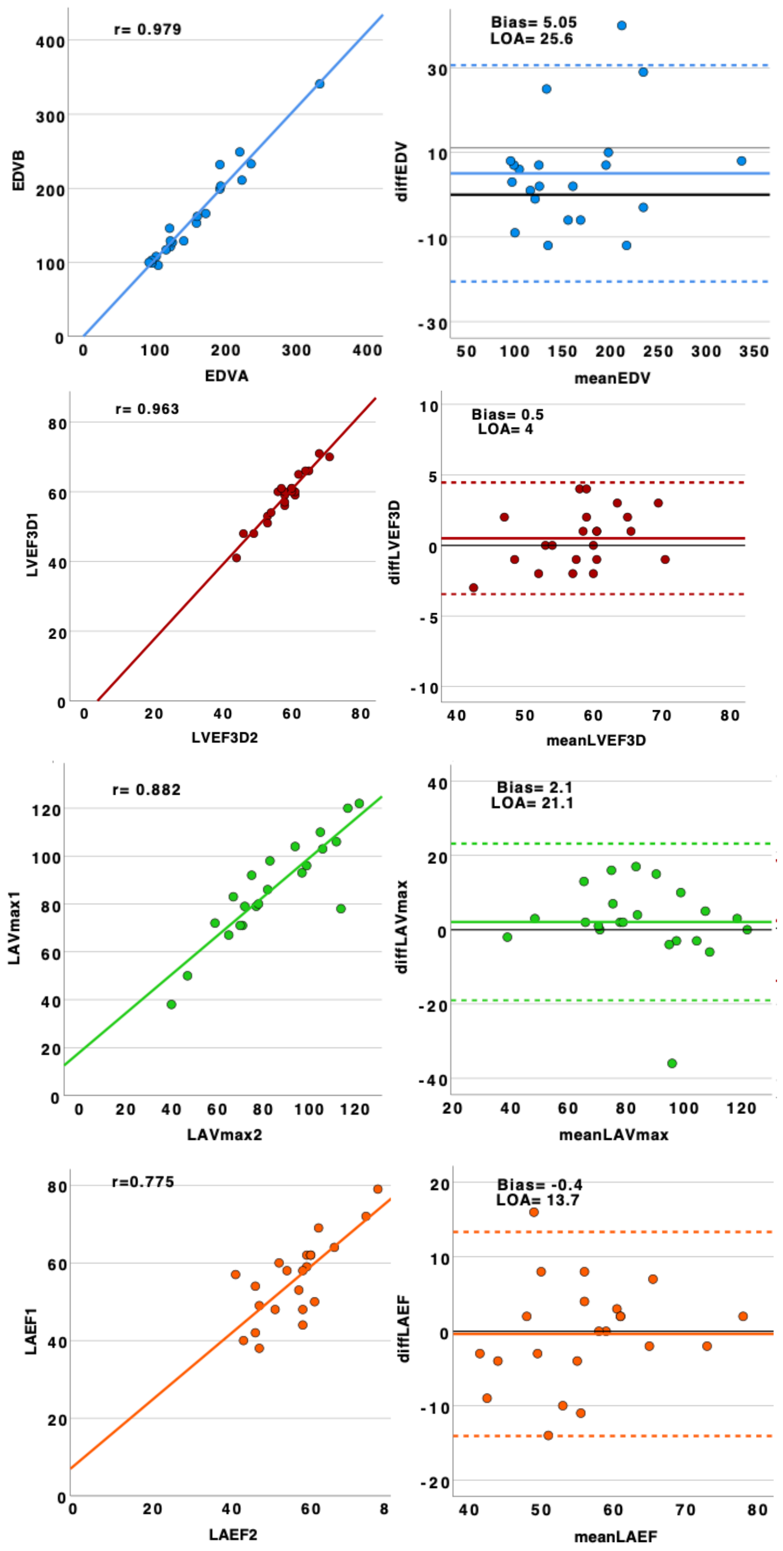

Legend: EDV= end-diastolic volume; LAEF= left atrial ejection fraction; LAV= left atrial volume; LVEF= left ventricular ejection fraction.

Figure S3: Inter-operator variability assessed by Pearson’s correlation and Bland-Altman Plot.

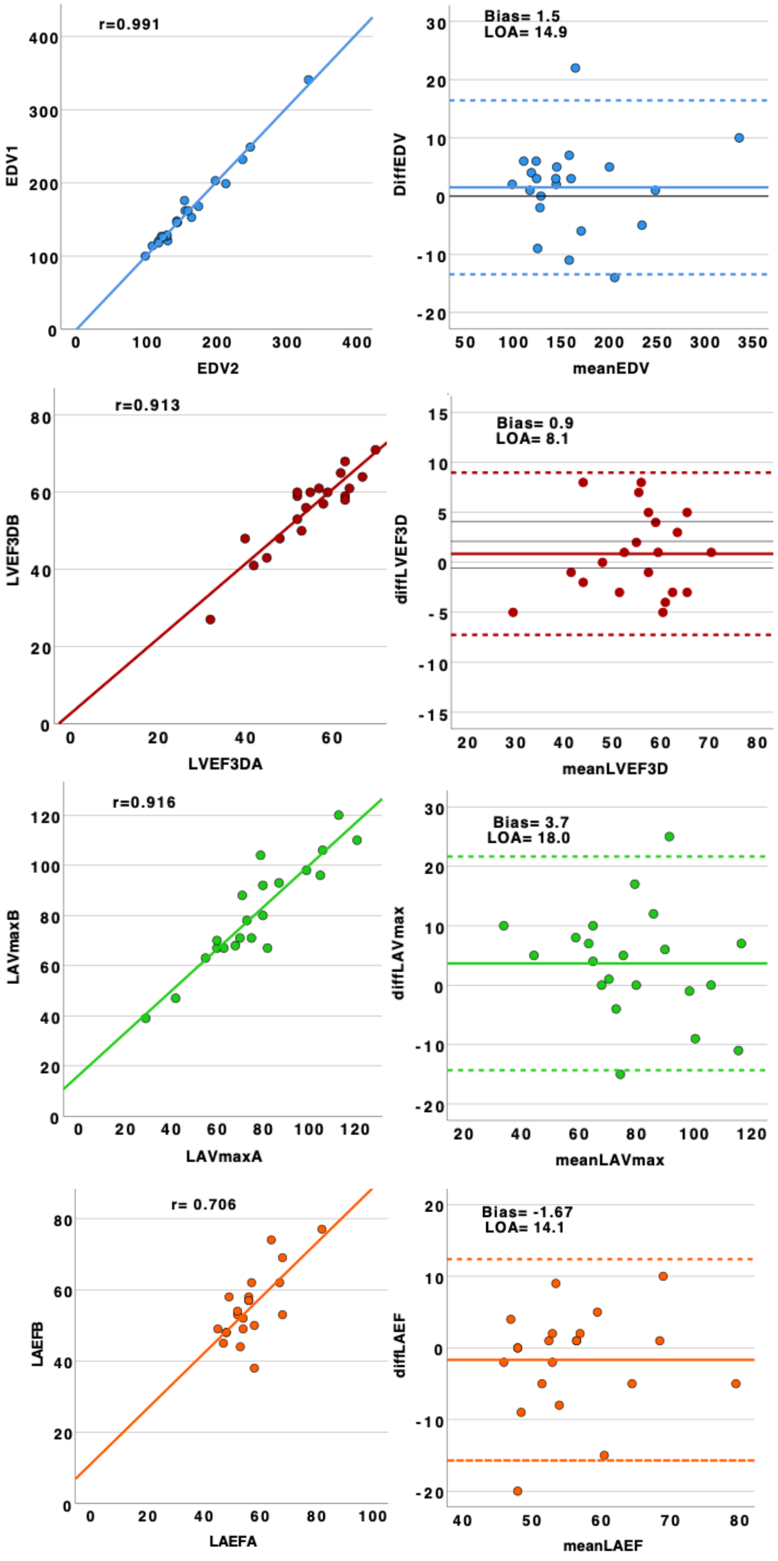

Legend: EDV= end-diastolic volume; LAEF= left atrial ejection fraction; LAV= left atrial volume; LVEF= left ventricular ejection fraction.
